# "The Hospital" Nursing Mirror

**Published:** 1900-02-03

**Authors:** 


### "The Hospital" Nursing Mirror.

Being the Nursing Section of "The Hospital."

[Contributions for this Section of "The Hospital" should be addressed to the Editor, The Hospital, 28 & 29, Southampton Street, Strand, London, W.C., and should have the word "Nursing" plainly written in left-hand top corner of the envelope.]

### Notes on News from the Nursing World.

### THE QUEEN AND THE BUCKINGHAM NURSING HOME.

The Queen has manifested her interest in the Buckingham Nursing Home by sending, through Lady Addington, a valuable quilt. In any circumstance the gift would be much appreciated, but the fact that the quilt was worked by Her Majesty greatly enhances its value. The incident affords yet another striking illustration of the sympathy which the Sovereign feels not only for those who are nursing sick and wounded soldiers in South Africa, but also for nurses engaged in what some persons choose to call hum-drum work at home. The Buckingham Nursing Home is pleasantly situated on high ground, and has two large wards, one male and one female, containing respectively five and six beds. There is also one private ward. The patients are admitted by subscriber's letter, or by weekly payments varying from 7s. to £2 2s. The staff consists of a nurse-in-charge and four other nurses, who do the work of the home and the district. Two, as a rule, are employed in the home, the others taking the district or private cases. Besides the town of Buckingham itself, visits are paid by the nurses to nineteen outlying villages when required.

### NURSING IN MARITZBURG MILITARY HOSPITAL.

A Sister of the Army Nursing Reserve writes us from a military hospital at Maritzburg: "I had a dreadful voyage out; gales and storms most of the way, and ill nearly all the time. When we got to the Cape two of us were ordered on to Durban, so we had another week on the sea, and then came straight on here. Fort Napier, the barracks, and usual military hospital belonging to it, are all turned into wards, but the sanitary arrangements are *nil*. No drains, and any water required is brought into the ward in a can. The buildings are some of brick, others wood, and others again are merely tent wards, but they are a good deal scattered and we have a lot of walking to do. Although the fear of the Boers attacking us is passed, we are still fortified to a certain extent; but the ground around the wards is no longer covered with the barbed wire, of which we were much more afraid on dark nights than of the Boers themselves. We have been kept dreadfully busy, and it is often such sad work. We have had the wounded down from Willow Grange, Estcourt, and last week from that awful battle at Chieveley and Colenso. One night I had about 15 stretchers in the ward at once, so that I hardly knew which way to turn. They were sent straight from the field—in many instances broken thighs done up with rifles as splints over the uniform, so that you can imagine the difficulties of getting the patients into bed.

### NURSING IN MILITARY HOSPITALS.

The "Surgeon-General," who has replied to some criticisms by Mr. Michelli, of the Seamen's Hospital, Greenwich, on the nursing of sick soldiers in military hospitals, does not dispose of the contention that more women are wanted in such institutions. Writing, we presume as the representative of the R.A.M.C., he, indeed, says that "We should not object to having a larger permanent proportion of nursing sisters." In these circumstances, and assuming that the expense of the additional proportion is a barrier, there is a good deal to be urged in favour of the proposal of Mr. Michelli, that "some of the money subscribed to the war funds might with advantage be expended in providing trained female nurses for the military hospitals." The orderlies are clearly necessary; but the point is that they are unduly numerous, while the nursing sisters are inadequately represented on the staff. In connection with this question, an interesting and suggestive report on nursing in military hospitals, by Dr. Groves, will be found in another column.

### A WARNING TO VOLUNTEER NURSES.

We continue to receive applications from nurses anxious to go out to attend to the wounded in South Africa, some of whom are willing to start on their own responsibility and at their own expense in the hope of obtaining employment at the front upon their arrival. A nurse on the spot, who till the outbreak of hostilities had been employed in Johannesburg, shows how futile these hopes are. As long ago as November 9th she went to Durban, with the view of getting to the front. She applied for leave to take the troops in the "hospital" ships round to Cape Town, but could not get the appointment; and later on was again disappointed when she expected she was going to be sent to the capital, although a surgeon-major was interesting himself in her behalf. In her last letter, written at Christmas, she says, "I am still waiting to hear from Maritzburg that I am wanted, but it may be a few more weeks before I get my call, as many nurses have come from home, and the 'Trojan' and the 'Spartan' had nurses on board."

### A CRY FROM AUSTRALIAN NURSES.

The nurses of Australia are asking that some of them should be sent to South Africa. Considering the number of Colonial troops who are fighting for the Empire the demand is not unreasonable. There is no doubt that medical and surgical nurses of capacity and experience could be easily selected in Australia, and all of us must sympathise with the plea that a wounded soldier from the Antipodes would be delighted to find himself tended by one of his own country-women. If the authorities can see their way to entertain the idea, we are quite sure that nurses in the old country would not grudge a few of their sisters across the seas the chance of being able to minister to the needs of their kinsmen, or to convey a last message to the loved ones at home.

### THE CASE OF THE ASSISTANT MATRON OF THE PARK HOSPITAL.

A futile attempt was made on Saturday by certain members of the Metropolitan Asylums Board to prevent Miss E. J. Atkins, assistant matron at the Park Hospital, from being allowed leave of absence in order to undertake the management of a hospital at Cape Town during the war. The opposition was based on the ground that to grant the permission sought would disorganise the whole of the internal economy of the Park Hospital; but as this view was only adopted by three members of the Board, it must be assumed that the overwhelming majority are quite satisfied that the absence of Miss Atkins will not have the results suggested. The first duty of the Board is, of course, to the patients under their care and the ratepayers whose money they are responsible for spending. But since Miss Atkins will draw no salary while she is away, and a temporary assistant matron will take her place without extra expense to the hospital, neither the one nor the other can very well suffer by the arrangement.

### NEW YEAR'S HONOURS FOR PLAGUE NURSES.

Sister Gladys and the Misses L. Green, M. Harvey, P. Leslie, and E. E. Hill have been created Honorary Serving Sisters of the Order of St. John of Jerusalem, in recognition of their services in plague nursing in India.

### THE INDIAN ARMY NURSING SERVICE.

It is proposed to increase the establishment of the Indian Army Nursing Service so as to provide sisters for several stations in the hills where no nursing sisters are now available. Three sisters are to be sent to Kasouli, a large sanatorium near Simla, which has hitherto had no nurses.

### THE NURSES' CO-OPERATION.

The report of the Nurses' Co-operation for 1899 is exceedingly satisfactory. The number of cases attended by the nurses was 7,048, or higher than that shown in any previous annual record, and the staff has been increased to 490 nurses. The addition of asylum-trained nurses has more than come up to expectations, and they have been constantly employed. The demand which the society has for these, as well as for general nurses, is usually far in excess of the supply. The new home—in the building of which the late Dowager Lady Howard de Walden took such an active interest—is fast approaching completion, and will add much to the convenience and comfort of the staff. A club-room and a restaurant will be attached, with ample bed and box room accommodation. Princess Louise and Miss Florence Nightingale have both expressed their satisfaction at the erection of a residential home for the use of the nurses, with separate sleeping rooms. When the home is ready for occupation applications from eligible candidates, which already exceed by some hundreds the number of nurses whom the committee are willing to enrol each year, will probably rapidly increase. The financial condition of the society is also extremely satisfactory. The gross receipts from patients amounted to £41,941, an increase of £3,792 on the previous year. Of this sum £38,790 was paid to nurses. These figures suffice to attest the value of the Co-operation to the nursing profession.

### THE DIFFICULTIES OF OBTAINING WORKHOUSE NURSES.

At a recent meeting the Newton Abbot Guardians had under discussion the difficulties of obtaining nurses for Poor Law work. It was stated in a return prepared by the clerk that other unions had gone through the same experience, but some of these institutions had minimised their difficulties by increasing the nurses' salaries and by making them progressive. They had also provided nurses' home apartments away from the workhouse. The committee then recommended that nurses who had completed two years' work should receive a gratuity of two guineas as a mark of appreciation of their services; also that the Board should subscribe to a lending library so as to provide books for their nurses. The Rev. C. Knibbs moved the adoption of this suggestion. Dr. Ley approved the recommendations, and said he agreed with the proposals made to brighten the lives of the nurses, as they had a most monotonous time. But he considered that the Board had missed the crux of the matter. "They would never get hospital-trained nurses to stay long in workhouses; the work was too uninteresting. He would like to see a strong recommendation to the Local Government Board in favour of workhouses containing 100 sick beds training their own probationers." But though the training of probationers in small union infirmaries might be useful in lessening the difficulties of Boards of Guardians in obtaining nurses, it might also be the means of introducing a very inferior certificated nurse, for it is a rare thing to get acute cases in the smaller union infirmaries.

### A WELSH-SPEAKING QUEEN'S NURSE FOR DOLGELLEY.

The committee of the Dolgelley and District Nursing Association have been fortunate enough to obtain the services of a Welsh-speaking Queen's nurse, who will take up her duties this month. It has hitherto been found impossible to secure a suitable nurse with this desirable qualification for work among Welsh people. We are glad to notice that, owing to the representations by Mrs. Franks, the inspector of Queen's nurses for Wales, who visited the district twice last year, it has been decided that patients outside a three-mile radius requiring the attendance of one of the nurses should provide a conveyance for her. Mrs. Franks suggested two miles. Possibly the compromise may be found to suffice, but three miles is a considerable distance for a nurse to be expected to have to walk to her duties.

### NURSES AND DIPHTHERIA.

At the annual meeting of the Londonderry District Nursing Society, over which the Duchess of Abercorn presided, the Lord Bishop of the diocese said "he knew no uniform, not even the glorious uniform of the British nation itself, more honoured than is the uniform of the cultured, educated, and refined ladies who are doing the work of nursing in Londonderry." In another passage of his speech he alluded to "those ministering angels who had gone among the people and inspired them with hope." Even more satisfactory testimony to their work than the Bishop's panegyrics is the fact that during last year 920 of 1,053 new cases were sent in by medical men, which proves how increasingly the doctors in the city are availing themselves of the nurses' services. But perhaps the most striking tribute was that of Dr. Byrne, who, referring to the success with which diphtheria had been treated, stated that "it was largely due to operations which the medical men dare not have attempted but for the assistance which the nurses rendered."

### ST. THOMAS'S CONVERSAZIONE.

On Friday a conversazione of a most enjoyable character was held at St. Thomas's Hospital for the nurses and their friends. The main lower corridor was decorated with curtains, flags, palms, &c., and a tastefully-arranged stage was erected in the main entrance hall, the whole being lighted by numerous Japanese lanterns and electric light. Refreshments were provided by the Treasurer, and served in the out-patients' waiting-room. Amongst the side shows were four palmists, who were extensively patronised; an X-ray dark room, where Dr. Blacke and his assistants exhibited an interesting collection of slides; Mr. Seligman's pictures and curios from Borneo; a pathological table furnished with microscope slides exhibiting specimens of the blood in various diseases; a physiological table; an instrument table, and mementoes of the Turco-Greek war in the shape of rifles and bullets. Great interest was excited by the dexterous manipulation of a glass-blower. The programme of band music was very good, and the exertions of the "Blue Boracic Band"—a troup of nine students of the hospital—were highly appreciated. The programmes were creditable to the artistic skill of the organisers of this pleasant social gathering.

### MAKING A START AT PONTYPRIDD.

A public meeting will be held next week in the fast growing town of Pontypridd for the purpose of discussing the proposed formation of a nursing association. Steps have already been taken, under influential local auspices, to initiate the movement, and a strong committee has been got together. The committee, which consists almost entirely of ladies, have decided to start with three nurses—one for general nursing and two for maternity cases; and it may be hoped that there will be a general readiness to uphold them.

### ONE OF THE REASONS WHY.

It will be remembered that a short time ago we published the fact of Nurse Norton's dismissal by the Workhouse Master from Tendring Union for refusing to bath male patients. We learn, not without surprise, that the Guardians have since experienced unusual difficulty both in obtaining and in keeping nurses. At one of their recent meetings it transpired that advertisements for nurses during the past half-year had cost the Board a considerable amount, and some of them said they were at a loss to understand why they could not keep their nurses in their service. It was then suggested that the salaries of the nurses should be raised, but a guardian pointed out that their salaries were as high as the average, and, in fact, in excess of those in most unions of the same standing. The suggestion was therefore allowed to drop. There is no doubt that the Tendring Guardians would find on investigation that one of the reasons why their nurses leave so frequently is the obnoxious rule which makes it imperative for them to remain in the bath-room during the bathing of male patients, and if they persist in appointing nurses who are ignorant of this rule, they must be prepared to put up with resignations when the latter discover their employers' idea of a nurse's duties.

### THE CHARWOMAN MIDWIFE IN IRELAND.

A correspondent sends us the following from a town in Ireland: "The charwoman not turning up on scrubbing day, I sent to inquire the reason, only to be told that she was engaged at a confinement case. When she did arrive, I asked her if she were a midwife. 'I am, indeed, miss,' she replied. 'Of course, I have none of those papers that the parish midwives have, but I'm at that trade for the last 25 years, ever since my husband went to the asylum, poor man. And 'twould do your very heart good, miss, to see the two fine boys (twins) I presented to Mr. —— last week. The people consider I'm a very lucky sort of a person, and that's why I get more to do than Mrs. ——' (a qualified midwife). 'Had you a doctor at this case?' I inquired. 'Not one, miss! I never yet had to call in a doctor only on two occasions, so that shows for itself how successful I am. Thank God for it!' I asked no more questions; but I cannot help wondering what is the use of going in for examinations, &c., and spending money, when a woman like this, who cannot even cook a little porridge, is allowed to do as she likes."

### SHORT ITEMS.

Miss M. Essex, formerly of Houghall Hospital, Durham, informs us that the local papers were mistaken as to the nature of her appointment at Guy's Hospital. She has only entered as a probationer.—A course of six lectures on "Some Main Issues in Social Work" will be given by Mrs. Bernard Bosanquet, at the Westminster Town Hall, on consecutive Fridays, at half-past eleven a.m., beginning on Friday this week. Among the subjects treated will be the Aid of Children, the Poverty of Old Age, the Housing Problem, and the Poor Law and Friendly Societies.—The Queen, in her far-reaching sympathy for the sick and ailing, directly she has pheasants to spare sends them to the patients at the hospital, as she has just done to the Cancer Hospital, Brompton, and the Hospital for Incurables at Putney. Lord Rothschild, it will be remembered, performs a similar act of kindness, but in his case it is the London omnibus-drivers who benefit.—Miss Napper, matron of the Surrey Convalescent Home for Men, at Seaford, has written to the Soldiers' and Sailors' Families' Association stating that she will be very pleased to receive Surrey soldiers and sailors on their return from the war.—Miss Ada Giles has written a spirited poem called "The Red Cross Nurse," which will probably be set to music, with the idea of devoting the proceeds of the sale to the War Fund.—A list of the midwives who passed the examination of the Obstetrical Society of London last month appears in *Nursing Notes* for February.—On Thursday a new nurses' home in connection with the Gateshead Nursing Association will be opened.—One of the exhibits at the forthcoming Earl's Court Exhibition will be a model hospital and convalescent home, in connection with the Charing Cross Hospital, erected to show how our wounded soldiers are treated and restored to perfect health.

### Lectures to Nurses.

By Carstairs Douglas, M.D., B.Sc.Edin., Professor of Medical Jurisprudence, Anderson's College Medical School, and Dispensary Physician, Samaritan Hospital, Glasgow.

### DIETETICS AND THE PHYSIOLOGY OF DIGESTION

(*continued*).

In the first stage of gastric digestion the ptyalin in swallowed saliva still continues to act as starch, a little lactic acid may be formed by bacterial action; hydrochloric acid is secreted, but does not become free (going into combination with the proteids of the food), and albumoses begin to be formed under the action of this acid and pepsine. The second stage of gastric digestion may be said to commence when the proteids are saturated, as it were, with hydrochloric acid, and some of the latter occurs free and uncombined. Then at once the action of ptyalin ceases, no more lactic acid is formed, and the energies of the stomach are directed to the making of albumoses and peptones out of proteids, the object of this transformation being solely for the purpose of rendering albuminous foods more easily absorbed into the system. Ordinary meat is not composed of pure proteid; the muscular fibres, of which it is constructed, are held together by white connective tissue, largely made up, in the raw state, of collagen. Now collagen on boiling is converted into gelatine, and this melting at body temperature allows the fibres of meat to fall apart more readily, and so to be more easily digested.

What is the effect of gastric digestion on milk? When milk enters the stomach a true curd is produced by the action of the rennin, due to the formation of insoluble casein which, entangling the fat-globules, forms the curd. Besides this effect from the action of the rennin, a partial precipitation of casein takes place by the hydrochloric acid of the gastric juice. The casein is finally converted into acid-albumin, then into albumoses, and finally into peptones.

What is the effect of the gastric action on fat? In the stomach, fat is highly indigestible, there being no ferment or acid there capable of acting upon it. It merely gets broken down into small masses and freely mixed with the rest of the food under the churning action of the muscles of the stomach. During all this time the pyloric sphincter has been relaxing at intervals, and eventually the whole of the acid chyme is passed into the small intestine. Here it speedily meets with the alkaline secretions of the liver and the pancreas, and the reaction of the fluid being changed pepsin at once ceases to act, its place being taken by certain new and very energetic ferments. Let us, before we consider these, reflect for a moment on what the composition of the chyme is, which has just left the stomach. (1) It contains dextrin, maltose, perhaps a trace of glucose, and some starch still unacted on; (2) it contains albumoses, peptones, unaltered proteids, and perhaps some gelatine, acid-albumin, and milk-curd; (3) it contains fats, as yet unchanged; (4) it contains water, salts, and free hydrochloric acid.

The first effect produced by the combined bile and pancreatic juice is the neutralisation of the free acid. Thereafter the following changes occur: (1) The powerful amylopsin, which can act even on unboiled starch, rapidly changes the starch in the chyme into maltose. This ferment is absent from the pancreatic juice of newly-born infants, which indicates clearly that they are not intended by Nature to have starchy foods—a view not always held by their mothers. (2) The ferment steapsin acts on fat in a twofold way: first of all, it aids mechanically in the formation of a fat emulsion, *i.e.*, a mixture where the fat-globules are uniformly disseminated in very minute size; and, secondly, it acts chemically by splitting up the fat into a fatty acid and glycerin, and this fatty acid, partly remaining free and partly converted into a soap by the alkali of the pancreatic juice, still further aids the formation of the fat emulsion. In this way fat is brought into a fit state for absorption by the intestinal villi. (3) The ferment named trypsin acts promptly and effectively on proteids which have escaped the action of pepsin in the stomach, converting them into peptones, and even going further and decomposing peptones into such bodies as leucin and tyrosin. (4) The milk-curdling ferment of the pancreas can produce a curd from milk in the same way that rennet does.

It may be asked at this point—If the pancreatic juice is so powerful, what is the use of the bile? In reply, we may say that bile has practically no digestive power of its own, but that it can assist that of the pancreas, and is of special value in the digestion and absorption of fats. This may be readily recognised clinically in cases of obstructive jaundice. Many nurses must have noticed how white the motions are in such cases, and may have (erroneously) attributed this to want of bile. As a matter of fact, the colour of the motions in health is not due to bile, and the clay-coloured appearance they present in jaundice is due to the presence of fat which has not been digested and absorbed on account of the want of bile.

To all intents and purposes, digestion is completed after the food has been acted on by the secretions of the pancreas and the liver; the only further change that occurs is the conversion of cane sugar and maltose into glucose or grape sugar by the action of the ferment invertin in the succus entericus of the small intestines. The object aimed at by all these chemical and physical processes is to bring the food into a fit state for absorption into the system. Proteids have been transformed into albumoses and peptones—although native proteids, as they are called, can also be absorbed as such; starch, cane sugar, and milk sugar have all eventually been converted into glucose; fats have been partly emulsified, partly broken up (saponified); water and salts require no special change. In the mouth and gullet absorption is practically *nil*; in the stomach there is little, if any absorption of water, but a certain amount of sugar, salts, and peptones find their way into the capillaries of the gastric mucous membrane, and are carried off viâ the gastric and portal veins to the liver, while the bulk of absorption takes place in the small intestines, a little also being accomplished in this way in the large bowel.

Albumoses and peptones, salts, sugar, and water pass through the intestinal mucosa direct into the blood-capillaries, and are conveyed by the portal vein to the liver, the sugar being stored up there as glycogen or animal starch. Curiously enough the albumoses and peptones are all changed during the process of absorption back again into the native albumins and globulins in which they originally existed, and it is in this form that they are found in the blood-stream. Fats are not absorbed directly into the blood-stream, but into the lymphatic system by means of small tributaries of the latter named lacteals, with which the intestine is richly provided. It is by means of the delicate little processes called villi that the fat droplets gain access to the lacteals. Each villus is a tiny club-shaped process, containing in its interior a lacteal offshoot from the lymphatic system. This little vessel is surrounded by a space containing free amœboid lymph-cells, while the outer lining of the villus is made up of columnar epithelium. In the absorption of fat the fine oil-globules pass through, not between, the columnar epithelial cells. It is probable that they are then seized upon by the wandering lymph-corpuscles and carried to the lacteal, and that they are then discharged into its interior. These lacteals open into larger lymphatic trunks, which eventually combine to pour their white, milky contents (now called chyle) into the thoracic duct. This vessel passes upwards to the root of the neck, where it opens into the jugular vein on the left side, and in this way allows the fat to reach the general circulation.

That part of the food which either cannot or is not digested, along with various decomposition-products, mucus, bacteria, bile-salts, &c., goes to form the fæces, which must be cast off from the body from time to time.

### St. George's Infirmary, Fulham Road.

### OUR REPRESENTATIVE EXCLUDED FROM THE COMMITTEE'S MEETING.

The meeting of the Visiting Committee of St. George's Infirmary, Fulham Road, was held, as announced, on Monday afternoon, but in spite of the fact that the charges made in our columns were to be the subject of inquiry a proposal that our representative should be admitted was rejected by the committee on the ground that their proceedings are "always private." The Visiting Committee have themselves to blame if the inference is drawn that they were afraid of publicity.

#### How the Charges Were Treated.

We learn from other sources, however, that the various charges made by our correspondent against the parish authorities of St. George's, Hanover Square, in respect to the treatment of the patients and nurses, were discussed, but that no resolution to make amendments in any single particular was arrived at. The prevailing sentiment expressed appears to have been that things are perfectly satisfactory as they are, and that it is a matter for regret that attention should have been called to them. The lady, who in her capacity of a visitor to the infirmary, had the public spirit to inform us of what she had seen with her eyes and heard with her ears in the infirmary, was asked a number of questions more or less irrelevant. One member of the committee wanted to know what "she would think of him if he came to her kitchen and listened to complaints from her servants?" as if there were the faintest analogy between a private person's kitchen and a public institution supported by public funds. The committee, it seems, were not of one accord as to the "advantages" of the mixed wards. It was urged that when the adults and children were separated the mortality among children was much higher.

#### The Absent Matron.

A noteworthy feature of the proceedings was the absence of the matron. She preferred, or the committee preferred, that she should be represented by a written communication, in the course of which, we are informed, she controverted some of the statements made as to the insufficiency of the nurses' food. But she did not expose herself to the ordeal of cross-examination; and the reply of the author of the charges against the parish authorities was that the testimony of many different nurses was in direct opposition to the assertions of the matron.

#### The Grievance of the Committee.

Finally, it was objected by the committee that our correspondent had written to The Hospital instead of putting matters before them. On that point the answer is conclusive. Complaints have been made to them without effect for six or seven years. In these circumstances she decided that it was hopeless to make any further appeals in the same quarter. The result of the proceedings on Monday abundantly justifies her conclusion.

#### Our Correspondent's Charges.

Under these circumstances we think that it is worth while to recapitulate the charges which were made by our correspondent in The Hospital of January 6th. Shortly they are as follows:—(1) That the patients' food is inadequate in quantity and badly chosen. (2) That the interval between the meals is in some cases excessive. (3) That there are no separate wards for children. (4) That the means of commusnication between some of the wards is defective, and involve risks to the patients. (5) That the nurses are over-worked, that their meals are inadequate, and that they have been obliged to wear damp clothes.

Having failed to convince the Visiting Committee that reforms in these matters are required in the interests of the patients and the nurses, we now appeal to the Board itself and ask in the name of the sick and infirm, who are practically voiceless in the matter, that the whole management of this infirmary be made the subject of a searching inquiry.

### The Nurses of the Imperial Yeomanry Hospital.

### PRESENTATION OF BADGES BY PRINCESS CHRISTIAN.

On Monday afternoon there was a considerable gathering in Lady Georgiana Curzon's pretty rooms at 20, Curzon Street, the occasion being the presentation of badges and certificates of the Army Nursing Reserve Service by Princess Christian to a number of nurses, most of whom have been appointed to duty in the Imperial Yeomanry Hospital. The Princess, who was attired in very deep mourning, but who removed her heavy veil before entering the drawing-room, was received by Lady Georgiana, and afterwards some twenty or thirty ladies belonging to the committee were introduced by the Duchess of Bedford to her Royal Highness.

The nurses were then called up one by one to receive the certificates, which were dated January 10th and signed "Helena," and the silver badges, which have a Geneva Cross in the centre. One nurse was subsequently heard to remark that the badge was not so large as she had expected, and was quickly met by the rejoinder, "Wait till you get it on; you will find that it looks huge." Each nurse was introduced to the Princess, and she addressed a few words to one or two with whom she was previously acquainted. To Miss Thomson she said, "I am glad to hear that you are going out, and hope you will keep well." To Miss Penrose, who has worked under her for nine years, and was one of her first two private nurses in 1892, she also made some congratulatory remarks. The ceremony was both simple and brief, and concluded with the reading by the Duchess of Bedford of a letter from Mr. Treves on the admirable work done by some of the nurses at the front.

"We had stirring times since last I wrote. I and the two nurses, after a little delay at Maritzburg, went direct to the front—viz., Frere Camp. They were the only women in a camp of 30,000 men. We reached the camp on December 11th. Just before the great battle of the Tugela on December 16th, we moved up to Chieveley with No. 4 Field Hospital. I was present at the battle. The nurses remained at Chieveley, which was, however, within reach of the Boer shells. The scene on the battlefield was beyond description. The heat was terrible, and the wounded suffered severely from thirst. The wounded all came to four field hospitals just behind the big guns. Here we attended to no less than 800 wounded. They were sent on as soon as possible, mostly to No. 4 Field Hospital at Chieveley. It was here that the nurses did such splendid work. Water was very scarce, and the heat extreme. . . . The nurses did not have their clothes off for two nights and were at work night and day. Miss McCaul gave away all her handkerchiefs, gave up her water-bottle and her mattress. . . . Just before we went to Chieveley two Army nurses joined us, and I expect these are the only four women who have really been at the actual front. I cannot speak too highly of the work they did. Many men will remember the nurses at Chieveley. . . . We came back here on Sunday, but we had no camp to go to, so the nurses slept on the floor of a looted house and I slept in a wagon. . . . I wish the women of England could know how these four nurses worked at Chieveley—worked until they nearly dropped, and did an amount of good work that cannot be estimated."

There was then an adjournment for tea, during which an animated conversation ensued. The costumes worn by the nurses were varied. Several appeared in private dress; others wore the uniform of their hospital, or of the Army Nursing Reserve. It has been decided to send out 40 nurses, including two superintendents, but the whole of them had not been selected on Monday, and no list of names was available. Those who had already been chosen might, however, almost have been identified by their radiant faces, while those who were still in suspense looked correspondingly anxious. Among the former, who sail for South Africa on Saturday, the 10th inst., are Sisters R. G. Rolleston, Ethel Keene, E. Leggatt, H. Foggo Thomson, M. M. Penrose, and A. A. C. Higgs, but a full list has been promised by the committee for publication next week.

### The Commercial Element in Nursing.

By a Matron of Experience.

In The Hospital "Nursing Mirror" of January 20th Miss Wedgwood, matron of the Royal Free Hospital, is reported as strongly deprecating "a tendency to make the ommercial element too prominent in nursing," and is said to have ffirmed that the work is "utterly ruined if it is looked upon om a purely commercial standpoint."

Whi entirely confirming Miss Wedgwood's dictum in one sense, there is another side of the matter which is apt to be disregarded by those who join in the fashionable adulation of unpaid labour and wealthy workers.

In a clever parody of Mr. Rudyard Kipling's poem, "The Absent-minded Beggar," appearing in the same issue of The Hospital, we are reminded of the danger of one-sided charity, and of looking at a subject from one point of view only.

With eyes fixed upon what appears to be the most interesting and fascinating we are apt to lose sight of the ordinary and commonplace.

### The Other Side.

It is well, therefore, occasionally to hear the other side of a discussion. These are days of stress and pressure; of overcrowded labour markets and underpaid work. The struggle for bare life is often terrible, especially to those who are accustomed to better things—well-educated, capable, and refined women, the daughters of the clergy, of officers of the army, of business and professional men, of those apparently fairly well off now who may have little to fall back upon in days to come.

### Ladies who Must Work.

These gentlewomen must all work, and work for money. They should all receive a salary that will keep them respectably in lodging, food, and clothing, and surely no one would grudge them a sufficient surplus for some recreation both for mind and body. Fair remuneration for fair work to those who really require such remuneration in accordance with that state of life to which it has pleased God to call them is what we plead for.

The lady who is "so very poor" on £200 or £500 a year that she "must add to her income" ought to be the employer of labour, not a seeker after posts that should be filled by those in want of bread and butter. For an inverse reason the domestic servant should not be encouraged to leave her pantry or her kitchen, nor the daughters or widows of small farmers or tradespeople their dairies or their shops, to enter a profession demonstrated by Miss Nightingale and other noble women to be best undertaken by the cultured and refined. It is to be feared that the social chances held out to such women by entering a profession where they may be mistaken for and treated as gentlewomen has no little to do with their ardent desire to "become a nurse," and leads also to endless deception and fraud.

### Questions for Miss Wedgwood.

"Charity begins at home," and the writer would ask Miss Wedgwood which of the following individuals would be likely, all things being equal, to make the most trustworthy nurse, the kindest sister, or the wisest matron.

1. The rich woman, who, from ennui or a want of an intelligent interest in the pursuits of cultivated people and the ordinary ways of doing good, leaves her surroundings for fresh excitement or pastures new in a hospital?

2. The "young person," whose mother keeps a little chandler's shop or goes out charing, who as district nurse may be possibly entertained at luncheons and "at homes" by the county families and aristocracy of the neighbourhood in which she may work, and who may end by marrying the parson or the doctor?

3. Or the daughter who leaves her home in order to be able to support herself, and so save her parents the anxiety and expense of her maintenance (and possibly add to the income as well) when they are old and feeble and the slender purse becomes more slender still by the demands of a long illness or the calls for comforts necessary to advanced age?

### The "Commercial Element."

Is it a "commercial tendency" or a just estimation of, and consideration for, the true feelings and real wants of others—qualities without which no one can be a good nurse—that leads such a woman to seek employment in a hospital?

Is it the "commercial element" or a reasonable and just view of the necessities of life that would prevent her from unnecessarily overtaxing her strength by doing more than one person's work, and would lead her to insist upon receiving a salary due to her? She glories in the fact that she "works for money." Is this "commercial spirit" to be denounced or commended? Can it in any way clash with the conscientious performance of duty? Nay; does it not rather proclaim a well-balanced mind, a kindly nature, and a tender conscience?

On reflection everyone must admit that it is the plain duty of every woman who works to see that she receives the just remuneration of her labour, not only on her own account but *pro bono publico*; and she is not true to her working sisters unless she discharges faithfully this obvious professional obligation. Does Miss Wedgwood call this the commercial spirit that utterly ruins nursing?

### A Terrible Alternative.

Virtue, unfortunately, in this world does not always bring its own reward, and we have to make it worth while for women to be virtuous if we wish to cure or mitigate a shameful and crying evil.

This is not a pleasant subject, but it is one that the thoughtful will do well to ponder, and "prevention is better than cure."

### The Essential Characteristics.

Again, the "commercial element," or, in other words, a thoroughly business-like way of dealing with matters, is absolutely essential to the well-being of a hospital, nursing institution, or home of any kind. Where there is a lack of this there is always a tendency to confusion, which opens the door to great carelessness, not to say positive dishonesty.

The new matron, who stops pilfering and waste, makes it "very uncomfortable" indeed to dishonourable and careless nurses and servants, neither is she quite popular with the local tradespeople whose weekly receipts she thus curtails. Negligent and easygoing committees, too, are not always very sensible as to the advantage of the cleansing of "Augean stables," which cannot fail to reflect discredit upon themselves, and out of sheer spite they are apt sometimes to vent their spleen on the head of the offending Hercules who has dared thus to disturb the peace of the wicked!

Without being a pessimist there is a commonsense acceptation of the Biblical declaration that "there is none righteous; no, not one," which one is bound to acquiesce in. Make dishonesty both in work and money impossible by strictly rendered accounts and careful supervision, and suspicion becomes unnecessary. To insist upon a strict rendering of equivalents, whether in goods, work, or money, is an essentially "commercial element" in a hospital, but it is obviously the only honourable way of conducting it or of spending the subscribers' money.

### Nursing in a Durham Colliery Village.

Perhaps there are few places in England where there is more scope to be found for the work of the district nurse than in our large collieries. At first sight a pit village is by no means beautiful. The long rows of low houses all painfully alike in streets near the pit, the absence of gardens, and the hideous arrangement of middens or ashpits at the back, are among the usual features. So, too, on the front doors are the roughly chalked figures, mostly 3's or 5's, which to the uninitiated are puzzling, though the man (or in some cases the old woman) who holds the office of "caller" understands them. They are the directions as to the time the inmates require calling in order that the men and lads may rise, put on their pit clothes, and be off to the pit. It is almost impossible "to sleep the callers," as they express it, though this does happen sometimes, and in consequence a shift is lost. Uninteresting, however, as the long rows of houses appear, when we remember that each one is a home with its human interests, and receive the hearty welcome always accorded to the nurse, that feeling quickly disappears. Nowhere is skilled nursing apparently more needed, both from the frequent occurrence of pit accidents, epidemics, &c., and the prevailing ignorance in nursing matters. There are still many collieries in Durham without a nurse, and many of those now working are mission nurses, sent by the canon-missioner of the diocese. Never shall I forget my first experience in a colliery, my difficulty in understanding Geordie and his wife, my astonishment at the huge fires in the houses, and my horror at the almost entirely naked men, calmly washing themselves in the kitchens, on their return from work, at the same time welcoming you in. On that occasion I remember, receiving the intelligence that a man had been "sore lamed that forenoon." I enquired the abode of the sufferer, and after a good deal of difficulty, and being exposed to many curious eyes, found him the victim of a heavy fall of stone on his shoulders and back. Fatal accidents occur only too often. Sad, indeed, then, is the scene as the dead body, black from the pit, is carried in to the wife and bairns from which he parted with the farewell—so seldom omitted—but a few hours before. Most of such deaths are caused by falls of stone, though a short time ago I had a case in which the pitman was poisoned by foul air, and only lived a few hours after being brought home, never regaining consciousness. In another recent accident the victim was crushed between the cage, in which the miners ascend and descend, and a sett of tubs, and though not a mark was to be seen outwardly, the internal injuries were such that he only lived a couple of days. When death occurs in the pit, work is stopped immediately for the remainder of the day, and the sight of men and lads returning home at an unusual hour is often the first announcement of a fatal accident. Accidents involving the loss of many lives seldom occur, though I have known three or four injured at once.

A miner's funeral is a sight to be remembered. The simple hearse is preceded by the colliery or society's band playing the Dead March, and the huge banner of the society, on which are often suspended the dead miner's tools. After, come the widow and children of the deceased; in a recent case an infant only a fortnight old was taken with them, and baptised in the crowded church before its father's funeral proceeded to the cemetery. Behind all is the tramp of many feet, as the miners, in their Sunday attire or funeral black, follow their dead comrade to his grave.

Perhaps the most trying feature in nursing pit accidents is the intense sympathy and excitement of the neighbours. The sick-room, directly the doctor's and nurse's backs are turned, is thronged with sympathisers, loud in their expressions of dismay and gloomy forebodings, quite unmindful of the sufferer's ears. Even in typhoid cases—although *the* fever, as it is commonly called by them, is much dreaded—the neighbours throng in, and I have seen on my arrival, in a tiny room, as many as six men sitting solemnly round a dying man, and the adjacent kitchen also crowded. Much respect, almost amounting to reverence, however, is paid to the nurse, in almost all cases, and in spite of many difficulties to overcome, her work is a happy one, perhaps because so much is needed. "Eh! honey, what would we do wi'out ye?" is often heard, and much gratitude is shown in words and touching little gifts.

A sad feature is the mortality among infants, in great measure due to the carelessness and ignorance of the mothers, especially in matters of feeding and exposure to cold.

Abominations in the shape of press beds, feather beds, &c., abound, and heavy wadded quilts (certainly most beautifully quilted), but utterly insanitary, are on every bed, sometimes two or three of them in lieu of blankets.

Many of the houses have no upstair rooms at all, and are very crowded. Baths for the miners are terribly needed, and when one takes into consideration the many difficulties of his life one often wonders that the pitman generally is such a good, honest fellow as he is, though terribly neglectful of religious duties, given to intoxication when the pay-week comes, gambling, &c.

Typhoid claims its victims every year. At one time I had as many as fourteen cases in the district to look after, besides others of different natures, and the work is usually heavy. Colliery doctors find this, for their patients, paying the small sum of sixpence or ninepence a fortnight, do not hesitate to send for them for the smallest ailment. One assistant doctor used to tell me that he frequently had over a hundred cases to visit in the day. Very trying, too, is the pitman's rooted faith in bone-setters for all fractures or dislocations, the wife's carelessness and disregard of infection, and the firm belief that they have, that cases of erysipelas can only be cured by charming. Cases of phthisis are frequent, and epidemics of influenza, and, about the month of August, of diarrhœa and English cholera, very severe.

The pitmen's houses as a rule are very clean. Twice a year at least their wives have thorough cleanings, and whitewash and paper and paint to their heart's content, the all-pervading coal dust and the big fires so constantly kept burning rendering this necessary.

When I look back upon the seven years it has been my lot to spend among the colliers, and think of my varied experiences, of some too who, as they beautifully express it, "have gotten away," and as, passing from house to house, I meet with many a familiar face, each with a nod or smile of welcome, I can say, paradoxical as it may seem (for no one can say that a colliery is outwardly beautiful), "the lot is fallen to me in a fair place." For, though rough in manner and plain in speech, the pitmen and their wives are to be depended upon, and they rarely forget a kind word or deed. Altogether, given a genuine love of nursing, a nurse's life in a pit village, among them and their canny bairns, as they call them, may be a truly blessed and happy one.

### Death in Our Ranks.

We regret to record the death of Miss Katharine Murphy, for nearly six years the loved and esteemed matron of the Border Counties Home for Incurables, Strathclyde House, Carlisle. She died in the home on January 23rd, after a suffering illness of three months, most patiently borne. Deeply religious and truly conscientious Miss Murphy was, says a correspondent, an admirable matron and a skilled nurse. She was unwearied in her care for and attention to the patients under her charge, and her gentle sympathy and tenderness won the hearts of all, the nurses and household staff being equally attached to her. Miss Murphy's death is also deeply felt by her many friends in Carlisle.

### Echoes from the Outside World.

AN OPEN LETTER TO A HOSPITAL NURSE.

Last week almost the opening words of my letter were: "The fear lest we should fail to drive the Boers from their stronghold of Spion Kop makes one sick with apprehension, because it means so much to us." And now even a worse fate seems to have befallen us. At the cost of a fearful loss of life we captured the point, held it for 24 hours, and then, to quote one of the correspondents, because "Mortal men could not permanently hold such a position," taking advantage of the dark night, our gallant fellows abandoned it to the enemy. Sir Redvers Buller in his despatch cites as one reason for this action the want of water, of which it was believed, before the storming of the kop, there was plenty at the top; but even had the springs been abundant, the fact that the enemy's machine guns were so accurately ranged that often 16 shells a minute fell in the unfinished trench—the only shelter of our troops—is sufficient to show the folly of trying to retain such a death trap. Also, the only side of the mountain—which is considerably higher than Snowdon—open to us was far too steep for it to be possible to get any of our own guns up, and the hasty fortifications which our men succeeded in erecting were not sufficiently strong to be of any real good. The news that all the troops had re-crossed the Tugela, and that, notwithstanding General Buller's words, we *had* turned back, very naturally came to us in England with crushing force. For when we heard that Spion Kop had been captured, we, too, like the inhabitants of Ladysmith, could in fancy almost hear the tramp of the relieving forces. It is now said that General Buller, in addressing his men after the retreat, impressed upon them that, because they had retired from the position, they must not think their work had been of no avail. In his opinion they had gained the key of the road to Ladysmith, and he hoped to be within the town in a week. The achievement of such a longed-for result would indeed be a cause for general rejoicing. The idea of leaving the garrison to its fate, as some military critics assert has become absolutely necessary, is heart-breaking, and would make us regret that it did not fall when the great assault by the Boers was made in December. But whatever else we have lost, we have not lost hope, and cheering news may reach us any day. It is certain that we do not hear of disasters directly they occur, for the loss of Spion Kop and the retreat across the Tugela was known in Paris on Thursday afternoon, but the War Office in London published no statement of how matters stood till Saturday morning. Perhaps when our authorities have good news to tell they will tell it more promptly.

---

Whatever mistakes have been made by the Government, whether in their policy or in the conduct of the campaign in South Africa, I think that most of us who are glad to stand outside the range of party politics will deeply regret the proceedings at the opening of Parliament on Tuesday. I am not alluding to the brief debate which took place in the House of Lords, though the reproaches which Lord Rosebery, admittedly without the reflection that so often changes a man's intentions—or those of a woman either—launched at the head of the Prime Minister seem to have been little deserved. The attitude of the Opposition in the other Chamber can scarcely, however, fail to enormously increase the difficulties of the Government in carrying on the war; and Lord Salisbury appears to most people who are much more concerned about their country's interests than those of any set of politicians, to have "hit the nail on the head" when he intimated that the attempt to pass a vote of censure on the administration would excite sympathy and enthusiasm at Pretoria. The spectacle of a member leading the attack on the Cabinet, in which his brother fills a prominent office, strikes outsiders as odd, though it is fair to say that Lord Edmond Fitzmaurice did his best to show that Lord Lansdowne is only one of a company of transgressors. But the point is that the position will be misunderstood by the Boers. They will not read the speeches of patriotic Liberals like Mr. Robson, who are as anxious as the most fervent Unionists that the war should be waged with determination to the end. They will only grasp the fact that the Opposition are trying to turn the Government out by means of a hostile vote, and they will then proceed to foster the idea that the British nation is divided, and that if a general election took place to-morrow peace would be concluded on the conditions of President Kruger. Of course, they will be utterly wrong, as they were when they thought we should not put down their rebellion; but it is a pity—is it not?—that at the gravest crisis in the experience of the present generation, the party machine should be permitted to darken understanding and obscure the issue.

---

The contrast at this moment between the English and the Chinese Empires is a very marked one. Here, both nominally and really, we have a woman at the head of the State, who, by her deference to the advice of her Ministers, and by the wisdom of her own actions, when action is necessary, has done so much to make England what she is to-day. In China, on the contrary, a headstrong and untruthful, albeit a clever, old lady is doing her utmost to keep her country from adopting any measures which would tend towards improvement and civilisation. The Emperor Kwang-Hsu has from the beginning of his reign been entirely under the thumb of the Dowager Empress, but having injudiciously allowed his predilection for certain of his *entourage* to become apparent, he has proved that even he is not sufficiently a nonentity for the over-reaching Dowager, who has succeeded in getting him deposed, and now, it is said, is having him slowly poisoned, lest any endeavour should be made to reinstate him. The little boy, who is nominally to occupy the throne instead of Kwang-Hsu, is only about nine or ten years of age. Therefore he will give no trouble, especially as his tutor is to be a tool in the hands of the Empress. As usual, rumours are current that Russian influence has been at work, but those who have lived in China any length of time are quite prepared to give the Dowager full credit for originating and carrying out any movement that seems likely to promote her own ends.

---

Those people to whom the London season means a great deal, either because it affords them pleasure or because it brings them gain, observe with sadness the signs of the times. The Devonshire House reception the night before the opening of Parliament has usually been an important function, attended by a great number, not only of persons directly connected with the two Houses, but by all the *élite* who happen to be in town. This year there was no gathering in Piccadilly. On Monday night darkness and dulness reigned where all should have been light and brightness, the Duchess, like most other women of England, feeling that merrymaking, under the present circumstances, was an impossibility. In the afternoon she had attended the distribution of the badges to nurses for the Imperial Yeomanry Hospital, a ceremony which harmonised far more with the fitness of things than social gatherings. A Lancashire Liberal Association has also postponed its annual soirée upon the receipt of the news from Natal, and everywhere society is as quiet as if Lent had already commenced. A stranger arriving in London would be struck at once by the number of women in mourning, and of men wearing hat-bands. In the neighbourhood of the clubs it is rarely that we see a high hat with the ordinary silk ribbon on it—significant evidence of the officers already lost in South Africa.

### The Princess of Wales's War Fund.

The list is now closed, but a final announcement of subscriptions will be made next week. Amount already acknowledged, £217 19s. 10d.

| | s. | d. | | s. | d. |
|---|---|---|---|---|---|
| Nurse Wall ... ... | 2 | 6 | F. A. ... ... ... | 5 | 0 |
| Nurse Robinson, P.F... | 2 | 6 | M. E. Taylor ... ... | 4 | 0 |
| M. P. Crighton, P.F... | 2 | 6 | M. Essex ... ... | 2 | 6 |
| Policy 3,619, P.F. ... | 2 | 6 | E. Pugh, P.F ... ... | 2 | 0 |
| J. S. Rothwell, P.F. ... | 2 | 6 | Nurse Tilley, P.F. ... | 5 | 0 |
| L. T. ... ... ... | 2 | 0 | F. J. Jackson ... ... | 2 | 6 |
| Nurse R. Simons, P.F. | 2 | 0 | H. Coope ... ... | 2 | 6 |
| H. Cooke, P.F. ... | 1 | 0 | E. A. Walker... ... | 5 | 0 |
| Policy 3,456, P.F. ... | 1 | 0 | S. Emmitt ... ... | 5 | 0 |
| Sister R. Smith, P.F... | 2 | 6 | E. Trinder, P.F. ... | 5 | 0 |
| Miss Cave, P.F. ... | 5 | 0 | C. Marshall, P.F. ... | 2 | 6 |
| Nurse Roberts... ... | 1 | 0 | Nurse Featon, P.F. ... | 2 | 6 |
| Nurse Butler, P.F. ... | 1 | 0 | A. P. Webb ... ... | 2 | 6 |
| Nurse Dawe ... ... | 1 | 0 | Nurse Norah ... ... | 2 | 6 |
| Nurse F. Smith ... | 1 | 0 | Mary Hopkins... ... | 2 | 0 |
| Collected by Nurse | | | Nurse Gatenly... ... | 2 | 0 |
| Riell, Ryde:— | | | F. E. M. Palmer ... | 1 | 0 |
| Miss L. M. ... ... | 5 | 0 | Nurse Redman, P.F.... | 1 | 0 |
| T. C. ... ... | 1 | 0 | C. Streeting, P.F. ... | 1 | 0 |
| M. F. ... ... | 1 | 0 | Policy 5,098, P.F. ... | 1 | 0 |
| A. F. ... ... | 1 | 0 | E. W. Goodworth ... | 1 | 0 |
| Mrs. B. ... ... | 2 | 6 | Nurse Wheeler, P.F.... | 5 | 0 |
| R. E. W. ... ... | 1 | 0 | Nurse Callaghan ... | 2 | 6 |
| A. B. C. ... ... | 1 | 0 | Nurse Ponton ... ... | 2 | 0 |
| H. H. ... ... | 1 | 0 | Nurse Kathleen, P.F... | 2 | 0 |
| E. W. C. ... ... | 2 | 0 | R. V. W. Monteath ... | 2 | 0 |
| A. H. B. ... ... | 3 | 0 | Nurse Tomlinson ... | 2 | 0 |
| A. N. C. ... ... | 2 | 6 | G. P., 5,401 ... ... | 1 | 0 |
| E. M. H. ... ... | 1 | 0 | Policy 5,649, P.F. ... | 1 | 0 |
| Policy 6,526, P.F. ... | 3 | 0 | | | |

### Private Nursing in Syria.

By a Nurse.

The English chaplain's little son was lying ill of malaria. A nurse was necessary for him—at any rate by night. I, a newly-arrived member of the nursing staff of a small English hospital near Mount Carmel, was told off to the Parsonage for the willing task.

As I entered my patient's room I saw, lying on a bed in a corner, a small sleeping form with flushed face and contracted brow. Two or three mosquitos buzzed around, as if upon mischief intent. That mosquitos cause malaria is now a recognised fact, but it was sad to think that so young a child should fall a victim to these pests of tropical and semi-tropical climes.

It is a curious experience—this private nursing in Palestine; and I will quote last night's programme. It will give a very fair sample of many of the cases which will probably fall to my lot. I came on duty at nine p.m., at which time the anxious parents retired for the night. The child's temperature was 103 Fahr. After a small feed of milk and water he went to sleep, and I was left alone with only the many night noises to keep me company. First came the uncanny yells of an owl overhead, the sound not unlike a child's cry. Next the horse in the stable below kicked about, and generally made manifest his presence. Then followed the barkings of the many masterless dogs that infest the town.

As night wore on I stole out on to the verandah to watch the glorious eastern sky. Never before have I seen stars so thick and brilliant, and I wished I had known the names of many. As it was, I was able to distinguish Orion's belt, the Great Bear, and also the Southern Cross. In front of the house flowed the Mediterranean; blue as a sapphire by day, but dark and stormy at night.

As daybreak approached the native servants awakened and kindled charcoal fires, upon which they prepared my breakfast, porridge and hot lemonade.

The little patient slept most of the night, only waking for his feeds of milk and water, and to tell me that Father Christmas had come all the way from England to fill his stocking!

About six a.m. the grey dawn appeared, and then commenced the sunrise, which, over the distant Galilean hills, is indeed an impressive sight. The shadows lifted one by one, and after a while the sun rose like a large ball of fire. Almost instantly the horizon became a blaze of light, and the hills around reflected the beautiful aurora—Mount Hermon on the east, now covered with snow, Carmel on the west, standing out with all its majesty.

One could not but feel awestruck, yet silently thankful for the privilege of such a sight. I returned to our patient, who after awhile woke up. His temperature was still high, and I sponged him before going off duty.

During the day large doses of quinine—per mouth and rectum—were prescribed for him, as well as constant tepid sponging. These, with milk and water diet, seem to form the basis of the general treatment for malaria in this part of the world, and with excellent results.

### Nasal Feeding.

Suggestions by a Nurse.

It would probably interest many nurses to receive a more detailed instruction on nasal feeding than is usually given in text books. Over the first attempt the chief thing needed is confidence: and, if the operator will only divest this treatment of that halo of mystery and danger which surrounds it, this is easily attained, for it is much easier to pass a tube into the œsophagus than to make a false pass into the trachea. The best instruments to use for the purpose are a Jacques rubber catheter, which should be as large as the nostril will admit to prevent a tickling irritation of the passage, and also to give the œsophagus a thicker body to grasp; and a glass funnel—for a funnel a glass syringe answers admirably. The fluid passed may be, of course, anything which the stomach is capable of assimilating, in suitable quantities, at reasonable intervals of time.

The tube should be first warmed and well lubricated—vaseline or glycerine are equally good for the purpose—and when introducing the tube into the nostril it should be remembered that the passage takes an upward and backward before a downward turn. With a little suitable manipulation the tube passes this turning easily, and it should then be pushed rapidly onwards—in fact, I think that it is in a quick passage that success lies, for if passed slowly the tube tends to irritate and induce coughing. Sometimes in nervous subjects, even in quite young children, a nasal obstruction is met with when the nostril is quite normal. This is a reflex contraction of certain muscles, and the only way to deal with it is by patient persistence to tire the muscle, so that when a momentary relaxation takes place, the opportunity of quickly passing the tube on may be taken before the muscles again contract. When the passing of the tube is accompanied by coughing or retching its course is often directed into the mouth; so the operator should make sure before pouring in the fluid that this has not been the case. Of course if cyanosis occurs the tube must be immediately withdrawn, but it is a mistake to suppose that this is an inevitable result of the tube lying in the trachea. The only way to settle any doubt in the matter is to wait until any coughing or spluttering which may be induced has subsided, and then to observe whether or not air is being actually breathed through the tube. The more or less forcible expulsion of air through the tube, as the result of atmospheric pressure at the one end and intra-thoracic pressure at the other, is easily distinguished from definite respiration through it. When the fluid is all passed the tube should be quickly withdrawn, for the same reason that it should be quickly passed. It is highly dangerous to withdraw the instrument whilst containing fluid unless the catheter is firmly pinched during the process.

### Nursing in Military Hospitals.

WITH reference to the question of nursing in military hospitals, which has been raised in our columns, a report was presented at the last meeting of the Parliamentary Bills Committee of the British Medical Association from Dr. Groves to the following effect :—

" MILITARY HOSPITALS.

" In some of the larger military hospitals, as Netley, the nursing is done, in part at least, by sisters of the nursing staff. In station hospitals the privates—known as orderlies —under the superintendence of the sergeants and corporals of the R.A.M.C. do the nursing.

" The recruits of the R.A.M.C. receive instruction in nursing, 'first aid,' and ambulance work, dispensing, and in cooking for the sick during a probationary period in one of the larger military hospitals, and they are drilled in a military sense. The orderlies are promoted after examination.

" The non-commissioned officers dispense, do all the clinical recording, and act as clerks to the hospital, their duties as such absorbing a large amount of time. Many of them are superior men, and some of them are said to have been trained as pharmaceutical chemists, and others for the medical profession, before they entered the service.

" The regulations provide for three meals a day, the first of which is taken about half-past seven a.m., the last about half-past five p.m. When on night duty the orderlies are not allowed nourishment or money in lieu of it. Night duty commences at half-past five, six, or half-past six, according to season, and is taken two hours on and four off, or three hours on and three off. The men on night duty take the day duty until five p.m., and are liable to be called upon to assist in cases of emergency, or if assistance is needed by the night nurses through the evening and night. In times of pressure—as during epidemics or when the staff is reduced by drafts—the strain upon the orderlies and non-commissioned officers is often very great.

"The military conditions which obtain in these hospitals oppress the whole system of nursing and hinder its efficiency. Everything is made subordinate to 'drill.' Attention to the equipment—furniture, utensils, and so forth—takes precedence of attention to the patients. The hospital orderly signs for and is made responsible for the equipment of his ward; and if at the weekly or monthly inspection any portion of it is broken or deficient, it must be replaced at his private cost. Each morning there is 'parade' in hospital, with general inspection, which occupies much time and hinders attention to cases of acute disease.

"The nursing system in military hospitals would appear to be singularly defective from the civilian's point of view. It is possible that after the war sweeping reforms in the army may occur; but, in any case, the whole subject of administration, and more particularly the question of nursing in military hospitals, should be inquired into and reform pressed for. "J. GROVES, M.D."

### Presentation.

ROYAL ASYLUM, MORNINGSIDE, EDINBURGH. — Mrs. Findlay, charge nurse in the Women's Hospital, has lately been presented with a handsome tea service and cake basket. The cake basket bore the following inscription : "Presented to Mrs. Isabella Findlay by her friends as a token of admiration and regard on completion of 26 years' service as the head nurse of the Women's Hospital, Royal Edinburgh Asylum."

### OUR CONVALESCENT FUND.

WE have to acknowledge the receipt of 10s. from E. C. N.

### Appointments.

CROYDON BOROUGH HOSPITAL.—Miss Annie Bond has been appointed Matron. She was trained at Westminster Hospital, and upon completion of her training took charge of the enteric wards at the Leeds City Hospital. She has since been matron of the City Hospital, East Liverpool.

HOSPITAL OF ST. FRANCIS, NEW KENT ROAD, S.E.—Miss Clare Crowther has been appointed Matron. She was trained at the Homœopathic Hospital, Bloomsbury, and for the last two years has held the post of matron to the Oriolet Hospital, Loughton, Essex.

KIRKCALDY FEVER HOSPITAL.—Miss J. Lawson Gilchrist has been appointed Matron. She was trained at the Chalmers' Hospital, Edinburgh, and for the last five years has been on the staff of the Royal Scottish Nursing Institution, Queen Street, Edinburgh.

HANWELL COTTAGE HOSPITAL.—Miss D'Arcy, of Malmesbury Cottage Hospital, who was appointed Matron, has, through unforeseen circumstances, since been compelled to decline it, and the committee have conferred the post on Miss May Rose, formerly of Stockport Infirmary and the Liverpool Hospital for Cancer and Skin Diseases.

COLNE COTTAGE HOSPITAL.—Miss M. A. Hodgson has been appointed Matron. She was trained at the Bristol General Hospital, where she was afterwards staff nurse. She has since been staff nurse and night superintendent at the Sanatorium, Hull, and Queen's nurse at the Institute, Gateshead. She holds a midwifery certificate, and the L.O.S. certificate.

LOWESTOFT CONVALESCENT HOME.—Miss Stephanie Harvey has been appointed Lady Superintendent. She was trained at Guy's Hospital. She has since been matron at Reynard Hospital, Willingham, Lincolnshire, and assistant matron at Easingwold Hospital. She has also been attached to the Metropolitan Convalescent Home, Walton-on-Thames, and for three years to the Yeovil District Hospital.

MERCERS' HOSPITAL, DUBLIN.—Miss Jessie Powell has been appointed Lady Superintendent and Matron. She was trained at Sir Patrick Dun's Hospital, Dublin, and afterwards became staff nurse in charge of the gynæcological ward, and staff nurse in charge of the fever ward. She has also been matron at the Small-pox Auxiliary Hospital at Kilmainham, and lady superintendent at the National Hospital for Consumption, Newcastle, Wicklow.

### Minor Appointments.

WHITCHURCH UNION, HANTS.—Miss Emma Tull has been appointed Assistant Nurse. She was trained by the Meath Workhouse Attendants' Association at the Royal Bucks Hospital, Aylesbury.

UNION INFIRMARY, ST. ALBANS.—Miss Alice Maud Culyer has been appointed Assistant Nurse. She was trained by the Meath Workhouse Attendants' Association at the Home for Invalids, Aubert Park, Highbury.

KEIGHLEY UNION INFIRMARY.—Miss Margaret Thomson has been appointed Charge Nurse. She was trained at Mill Road Infirmary, Liverpool, and has since been charge nurse at Mill Road Infirmary, Liverpool, and district nurse.

MERTHYR GENERAL HOSPITAL.—Miss Jean Campbell Walkinshaw has been appointed Charge Nurse of the female ward. She was trained at the Swansea General and Eye Hospital, and was subsequently staff nurse in a large male surgical and accident ward, private nurse at the same hospital, temporary matron, and sister at the General Hospital, Llanelly; private nurse to the Swansea and South Wales Nursing Institute, Swansea.

### The Nurses' Bookshelf.

[We invite Correspondence, Criticism, Enquiries, and Notes on Books likely to interest Women and Nurses. Address, Editor, The Hospital (Nurses' Book World), 28 & 29, Southampton Street, Strand, London, W.C.]

Surgical Ward Work and Nursing. By Alexander Miles, M.D., C.M., F.R.C.S.E., surgeon to the Leith Hospital. With 333 Illustrations. Pp. 288. Price 3s. 6d. Second Edition. (The Scientific Press, Ltd., 28 & 29, Southampton Street, W.C.)

It is with no small degree of pleasure that we welcome the second edition of this invaluable work dealing with surgical nursing on the most enlightened principles. The market is somewhat overcrowded with text-books on the general details of nursing; surgical nursing, with all its modern significance, being left out altogether in the cold, or receiving but a cursory glance at best. Here, however, we have in clear, well arranged form, explanations, definitions, and recommendations of every detail concerning modern surgical operations. Section I. describes the principles of antiseptic surgery, the management of a surgical operation, and the appliances usually required. Lotions and their strength, their uses, advantages and disadvantages, form a very interesting chapter, and the rules laid down for surgical cleanliness of the hands of surgeons, nurses, and others should be committed to memory. Chapter II. on aseptic surgery is, perhaps, one of the best in the book. Section II. treats of the special nursing of surgical operations, and is admirable in its completeness. No detail, however small, has been overlooked, and the directions are so clear that the least intelligent may understand them. Ice, which used to be the only acknowledged remedy for chloroform sickness in the eighties, is now found to produce flatulence, and consequently is not to be recommended in abdominal cases. We are told that sips of hot water are preferable and also are more effectual in quenching thirst. We cannot help regretting, however, from a nurse's standpoint, Dr. Miles' wholesale condemnation of poultices. They may not, according to modern principles, be aseptic, but no one who has suffered acute pain could fail to testify to their comfort, for the relief of which they are far and away ahead of fomentation, as they retain the heat so much longer. Chapter XX. contains some valuable remarks on the treatment of burns and scalds. Picric acid and ichthyol are recommended as good dressings, and infinitely preferable to the oily and greasy applications which used to be so much in favour for such cases. Bed-sores may be treated antiseptically or with balsam of Peru and eucalyptus ointment, stress being laid on the nurse's duty in taking preventative measures. Section III. deals with the use of rest in surgery and the preparations which are required in connexion with it. All the various kinds of splints come under this heading, and the illustrations are excellent. The method of applying a Scott's dressing is fully described, and likewise the materials required for its preparation. Bandaging comes under Section IV., and is most exhaustively described, illustrations being given of all the more uncommon kinds. After a careful perusal of this excellent little book we have no hesitation in placing it in the very foremost rank among nursing text-books, and would like to see it in the hands of every nurse who is interested in the more scientific side of her profession.

The Englishwoman's Year Book for 1900. Edited by Miss Emily Janes, Secretary to the National Union of Women Workers of Great Britain and Ireland. (London: Adam and Charles Black. 1900. Price 2s. 6d.)

This is the twentieth year of the issue of this most valuable book of reference with regard to women's work. What "Whitaker's Almanack" is to the general public "The Englishwoman's Year Book" is to womankind. Every year sees it enlarged, and in this volume there are sections on education, employments, professions, science, literature, art, music, sports, pastimes, and social life, public work, philanthropy, and temperance, with lists of homes and charitable institutions, and an article on religious work. It is impossible to glance through the well arranged, concise pages of this book without being struck with the mass of information it contains. The articles and sections are the work of a variety of authors, most of whom are well known in relation to the subjects upon which they write. Some of these interpolated articles are really very interesting and full of information. Lastly, the whole of the book is carefully indexed, and so rendered available for use in its most convenient form.

The Zenana; or, Woman's Work in India. The monthly magazine of the Zenana, Bible, and Medical Mission. Vol. VI., 1898-99. (London: S. W. Partridge and Co., 9, Paternoster Row. Price 2s. 6d.)

The annual volume of "The Zenana" is full of interest for anyone who desires to keep in touch with the work being done for the women of our Indian Empire. While special attention is given to the work done in the mission hospitals there is no tendency to belittle the usefulness of the Government hospitals and those that are associated with the name of Lady Dufferin. At the same time it is made clear that in the opinion of the various writers who contribute their experiences to the volumes, the influence of Christianity alone can break down the innumerable barriers that exist to the full civilisation of India. The selfishness and hardness of heart, as well as the superstition that seem to be the offspring of heathenism come out in anecdotes told by one missionary and another from their own experience, and cannot fail to impress on all who read the need for mission work. Some useful hints are given by several lady missionaries, medical and other, as to the gifts most suitable for sending out to their protégés. Miss Haskew, M.D., complains that of the garments sent out to her many are far too elaborate for the recipients. Frilled nightgowns, she says, look utterly out of place on a Hindu or Mahommedan woman. She asks for very plain ones, open all the way down, and fastened with tapes rather than buttons. Nightingales also should be made plain and without ribbons. Certainly a saving could be effected in that way, and more articles sent out at no greater cost. She also asks for coarse, warm, woollen stockings, which perhaps no one ever thought of sending to India, but which are useful for keeping the extremities warm in operation cases. Those interested in the work of missions, and especially medical missions in India, cannot do better than consult the pages of "The Zenana."

"Only Joe; or, Short Tales of Homely Hearths." By I. E. Cutcliffe, Author of "The Old Motto," &c. (London: Skeffington and Son, Piccadilly, W., 1899. Price 3s. 6d.)

This little volume is published on behalf of the Children's Hospital in Great Ormonde Street, the profits arising from its sale being destined by the author to help the building fund of that useful institution. For this reason alone we might reasonably commend it to the kindly notice of our readers, but fortunately we can do so on its own merits, without regard to the object for which it is published. The stories are simple, and for the most part pathetic, but are told with a light touch, so that they never become depressing, and are illumined by that faith which looks beyond the darkness of the present into the reward that lies beyond for unselfish and noble lives. Some of the stories are by no means destitute of humour, notably the one dealing with a literary swindle, which one may surely venture to surmise is a personal experience, and all are told with a touch of sprightliness and imagination that make them pleasant reading.

### Everybody's Opinion.

[Correspondence on all subjects is invited, but we cannot in any way be responsible for the opinions expressed by our correspondents. No communication can be entertained if the name and address of the correspondent is not given, as a guarantee of good faith but not necessarily for publication, or unless one side of the paper only is written on.]

### CHRONICS AND CONVALESCENTS.

"A CONVALESCENT NURSE" writes: I wonder if enough attention is paid in hospital to the chronics? I was once warded in hospital myself with a chronic knee for several months. Everyone was most good and kind to me, but I was very miserable and depressed at the thought of giving up my work, and I remember I was silly enough to notice when sister did not ask me in the morning how my knee was. Of course, I know quite well that there was really very little difference in my knee from one day to another, and, now I am using it again, I feel very much ashamed of myself for being so depressed and silly. But if a nurse—who should know better—felt like this, I wonder how those who don't know better feel. I think a little more sympathy with our chronics would go a long way. I know that the acute cases must be attended to, and also I know how very little time one gets in the rush of the ward work; but if we could spare five minutes now and again with some of the convalescents and chronics I do not think it would be lost time. Let them know you take an interest in them. Do we realise what it means to lie month after month knowing and feeling we are not getting much better? That we have had to give up our work, and that there is small chance of getting it again after months of illness? It is so difficult for a strong, healthy woman to know quite how a convalescent, all nerves and irritability, feels, and how the least jar and noise will rub such a one the wrong way. I think that our hospital nurses of the present day are splendid in a great many ways. They get through a tremendous amount of work; they are smart, neat, and quick; they attend to every comfort of their patients; but there is often one thing lacking—the one little gift of sympathy, without which no nurse is perfect.

### DANCING IN HOSPITALS.

"A MATRON OF A HOSPITAL" writes: As the subject of dancing in hospital has come before the readers of your paper during the last two weeks, may I express my strong opinion that they are utterly out of place, and that all those who care for securing a high standard of nursing and efficiency of discipline will combine in effort to uproot what is detrimental to the furtherance of that object. It is recognised that strictly professional manners between the doctors, honorary and resident, is the *sine qua non* of a well-trained nurse, and all those who are responsible for the training of their nurses must, I think, regard any tendency to break down those essential barriers as detrimental to the good of the whole nursing profession. Any lack of discipline in a hospital must result in inferior nursing; the patients will be made the second consideration, and where that is so it must necessarily tend to the deterioration of the nurse's character and training. Self-control and the subservience of our own personal pleasure for the general good should be our aim, and the strictest discipline when rightly understood as an instrument for furthering that end would be more fully contested for and upheld.

"MATRON" writes: I can heartily sympathise with the matron of Lambeth Infirmary in her opposition to the perpetuation of a dance for the nurses. In my experience these nurses' dances have done the greatest harm both to the discipline of the hospital and to the care of the patients, which ought to be the first object. Instead of the relations which ought to exist between doctors and the nurses working for them, there is engendered a familiarity which leads to gossip, any fresh orders from superiors being discussed, and in many cases derided, the result being disloyalty and all the evils consequent, which re-act on the originators, illustrating the proverb, "Familiarity breeds contempt." Then those nurses who have the moral courage to keep apart have the finger pointed at them, and are laughed at as being "good." I should like to ask if the nurses in hospitals where these dances are not allowed are less contented and happy than in the others. In my experience it is quite the opposite, and I am thankful to say that I was trained where we never thought of such things inside the hospital, and the discipline there was the strictest. Of course I consider it the best managed hospital in London. Perhaps if there were less of this frivolous element encouraged among nurses inside hospitals and infirmaries we might meet less often with the class of giddy women who disgrace our noble profession, and who have taken up nursing because they wanted a "lively time," or perhaps to get married, &c. For what purpose should women take up training? Ought it not to be solely to learn intelligently the care of the sick and suffering, and if they want recreation of the kind they can get it elsewhere than inside institutions, which are supposed to be only for the ease and comfort of those ill and dying. Surely dancing inside such places is most incongruous. This subject comes up almost every year, showing the trouble caused. When committees see it in a proper light they will only allow hospitals to be used for their right purpose, and the difficulties of a matron who conscientiously seeks to train her nurses will be considerably reduced.

### THE COLONIAL NURSING ASSOCIATION.

MISS MARY KINGSLEY writes: When I received this morning a cutting from your valuable journal headed "Miss Mary Kingsley's Mistake," I thought, which of them? and read it in hope of receiving assistance in the form of correction. Alas! I have not got that assistance. I am perfectly well aware of the magnitude of the debt we all owe Mrs. Piggott in the matter of the Colonial Nursing Association. I have stated it freely and frequently. You will find it in print in the appeal I wrote for this association in "The British Empire Review." But in the address you are referring to, given at the Livingstone Exhibition, I did not refer by name to Mrs. Piggott, or, for the matter of that, to Dr. Patrick Manson, who has done similar work for the scientific study of tropical disease to that done for trained nursing in the tropics by Mrs. Piggott. I was on that occasion merely referring to Mr. Chamberlain's statesmanship in incorporating such things into practical politics. I have a great admiration for Mr. Chamberlain for his action in this matter. I sincerely wish he who has had the sense to call in science to combat the waste of white men's lives in the tropics would go further on the same road and call in science in the matter of saving black men's lives, and placing our rule over them on a basis from which a healthy development would be possible. Still, I never for one moment meant to give anyone the idea that I regarded Mr. Chamberlain himself either as an excellent trained nurse or an excellent scientific medical man. My address at the Livingstone Exhibition is in the hands of the printers, and when I get a copy of it I will send you one, hoping you may aid me by pointing out other mistakes I have made in it beyond that of not mentioning Mrs. Piggott, Mr. Chamberlain, Mrs. Antrobus, and Dr. Patrick Manson, Dr. Ross, &c.

### THE MONOTONY OF PRIVATE NURSING.

"AN ADMIRER OF FLORENCE NIGHTINGALE" writes: How I long to go out to Africa and help in the arduous and trying duties of nursing our wounded and dying brave soldiers. My longings are in vain as I am in a private nursinghome at present and cannot leave without notice. It seems to me that we nurses on private cases have such a number of uninteresting duties to perform that we simply get worn out at it, especially when we get patients whose sickness is mostly imaginary. We have seen so much genuine suffering in our probationer days of hospital life that we find a difficulty in nursing such patients. Here we are listening to their imaginary pains and aches from morning until night or from night until morning. The latter is preferable as there is a lull in the conversation during the hours of sleep. I wish doctors were more outspoken in treating such cases and would do something as one doctor I knew did. He told his patient to put on an apron and go into the kitchen for several hours each day. These directions did not please the patient, and therefore she asked the doctor not to call again. If some of these patients would visit the hospitals and study the sufferings of the many inmates they would surely come to the conclusion that they themselves were great frauds with their nurse to wait upon them to humour them in all their fads and fancies, and I am must say not always willingly. It is cases such as these which make us long to go to the front for real hard work.

### For Reading to the Sick.

"And He cast out the spirits with His word and healed all that were sick."

"Himself took our infirmities, and bare our sicknesses."—*St. Matt.* viii. 16, 17.

There is no sorrow, Lord, too light
To bring in prayer to Thee;
There is no anxious care too slight
To wake Thy sympathy!
Thou Who hast trod the thorny road
Wilt share each small distress;
The love which bore the greater load
Will not refuse the less.
There is no secret sigh we breathe
But meets Thine ear divine,
And every cross grows light beneath
The shadow, Lord, of Thine.

—*Jane Crewdon.*

Find thy reward in the thing
Which thou hast been blest to do,
Let the joy of others cause joy to spring
Up in thy bosom too!
And if the love of a grateful heart
As a rich reward be given,
Lift thou the love of a grateful heart
To the God of Love in Heaven!—*Macdonald.*

"Himself took our infirmities, and bore our sicknesses"; implying, apparently, that His miracles of healing were wrought, to some extent, by a transfer of some kind to Himself of the infirmities from which those afflicted with them were relieved; or, at least, that, for some mysterious reason, beyond our understanding, He could not have healed bodily troubles without making Himself subject to them; that is, by taking on Him our nature, with its liability to infirmities, and I would point out that the principle is obviously true of sorrows of the mind and griefs of the soul. Sympathy, in its exact meaning—actually feeling *with* another—has much to do with the healing of these. And the sympathy of God is what is assured to us by this vision of the question.

He took upon Himself the present temporal griefs and sorrows of humanity, from whatever source arising, tasting each and all; that, being acquainted by experience with them all alike, He might perfectly sympathise with, and heal by sympathy, the sufferers from them, to the world's end. . . . Oh, you who are men and women of sorrows, not unacquainted with grief, take your sorrows to Him, and make Him acquainted with all your griefs. So will you, at the same time, gladden His heart and find solace for your own. Especially go to Him with that godly sorrow that worketh repentance. And your life on earth shall be freer from distress, and in the end you shall share the blessedness He has won by His sorrows for Himself and all His. . . . Let His awful sorrowfulness, His unutterable grief, move us to give ourselves wholly to Him.—*Bourne.*

But if impatient, thou let slip thy Cross,
Thou wilt not find it in this world again,
Nor in another; here, and here alone,
Is given thee to suffer for God's sake. . . .
. . . If He should call thee from thy Cross to-day,
Saying, *It is finished!*—that hard Cross of thine,
From which thou prayest for delivrance,
Thinkest thou not some passion of regret
Wouldst overcome thee? Thou wouldst say, "So soon!
Let me go back and suffer yet awhile
More patiently! I have not yet praised God."
And He might answer to thee, "Never more;—
*All pain is done with!*" —*Hamilton King.*

### Notes and Queries.

**The contents of the Editor's Letter-box have now reached such unwieldy proportions that it has become necessary to establish a hard and fast rule regarding Answers to Correspondents. In future, all questions requiring replies will continue to be answered in this column without any fee. If an answer is required by letter, a fee of half-a-crown must be enclosed with the note containing the enquiry. We are always pleased to help our numerous correspondents to the fullest extent, and we can trust them to sympathise in the overwhelming amount of writing which makes the new rules a necessity.**

**Every communication must be accompanied by the writer's name and address, otherwise it will receive no attention.**

*Nursing.*

(179) Would you let me know how to become a nurse in one of the London hospitals, and (2) whether there is any age limit for the same? —*Norma.*

Write to the Matron of the Hospital, or see "The Nursing Profession: How and Where to Train," price 2s., from the Scientific Press. 2. The age limit varies.

*Home for Young Girl.*

(180) Could you or some of your readers recommend a home or institution where a young girl of seventeen could be placed? She is not capable of getting her own living until she has had some kind of training and supervision by some lady who would try to improve her and make some allowance for her. She is an orphan, but her sister, who is a trained nurse, would see that she was comfortably clothed, and would not require any wages for her until she was of real help.—*Anxious.*

It would be easier to help if "Anxious" had given more particulars as to the girl's capacity. If something superior to domestic service is aimed at see "The Englishwoman's Year Book for 1900" (Adam and Charles Black, publishers), price 2s. 6d.

*Rochester.*

(181) Will you kindly give me any information possible concerning the St. Bartholomew's Hospital, Rochester? I am anxious to know whether the training is good; whether probationers receive three years' training in the hospital wards; whether the nurses are comfortably provided for; also, whether it is a general hospital or not.—*M. A. L.*

St. Bartholomew's Hospital, Rochester, is a small general hospital of 72 beds. Apply to the matron as to the arrangements for the nursing staff.

*Nursing Reserve.*

(182) I should feel much obliged if you will kindly inform me as to where I should apply with a view to being enrolled as an Army Reserve nurse; (2) also what steps a nurse should take in order to become a Naval Reserve nurse.—*Irish Nurse.*

Apply to the Hon. Secretary, Army Nursing Reserve, 18, Victoria Street, W.C. 2. There is no Naval Nursing Reserve.

*French.*

(183) I am a trained nurse, and desire to learn French. Would you kindly tell me how to acquire it, self-taught?—*A. M. J.*

For self instruction the only thing is to work through a simple course of grammar and translation. Any good bookseller will advise you as to the book best suited for use without a teacher.

*Obesity.*

(184) A few months ago the name of a doctor who makes a speciality of the treatment of obesity was mentioned in The Hospital. I hope it will not be against your rules to give me his name and address. 2. Of what does the Salisbury treatment consist, and against what disease is it directed? 3. How should an intermittent pulse be counted for a full minute and for one only? Is it of any import to record the fact that it is intermittent, how many beats are dropped, and whether at regular or irregular intervals?—*Fairhazel.*

1. The address of all medical men can be found in Churchill's Medical Directory. 2. The Salisbury treatment is a system of dieting which should not be undertaken except under medical advice. 3. In taking the pulse it is well to note every peculiarity and to feel it at short intervals until approximate results are obtained.

*Nursing.*

(185) Could the Editor of the "Letter Box" help me as to starting as a visiting nurse? I was five years in hospital, &c. 2. What locality would he recommend me to take a house in?—*Anxious One.*

We regret that we are unable to recommend a locality, or to help *Anxious One* in her difficulty.

*Acme Chimney Cleaner.*

(186) Will any one kindly tell me if they have used the Acme Chemical Chimney Cleaner in an invalid's room, and with what success?—*Private Nurse.*

This seems to answer tolerably well with fairly clean chimneys; it does not, however, rival the sweep's brush.

*Homes for* [illegible]

(187) M. A. Hall's compliments to the Editor, an she would be glad if he could inform her of a ladies' home suitable for a lady, aged 77, where an annuity of £30 would be accepted for her maintenance and comfort during the remaining years of her life.

The Woodside Home, Whetstone, N., might be suitable.

*Standard Books of Reference.*

"The Nursing Profession: How and Where to Train." 2s. net.
"The Nurses' Dictionary of Medical Terms." 2s. 6d. net.
"Burdett's Series of Nursing Text-Books." 1s. each.
"A Handbook for Nurses." (Illustrated.) 5s.
"Nursing: Its Theory and Practice." New Edition. 3s. 6d.
"Helps in Sickness and to Health." Fifteenth Thousand. 5s.

All these are published by The Scientific Press, Ltd., and may be obtained through any bookseller or direct from the publishers, 28 & 29, Southampton Street, London, W.C.

### Travel Notes.

By Our Travelling Correspondent.

### XLIV.—ENGLISH WINTER SEASIDE RESORTS.

Before resuming our more detailed accounts of continental places suitable either for prolonged residence or for holiday-makers, I want to draw your attention to a few spots in England more or less agreeable in mid-winter. Unfortunately, we cannot enjoy the continental sun; that is a want that must be felt in our otherwise almost perfect island.

### The Cornish Coast.

The weather is usually comparatively mild on the south coast of Cornwall. You will see by a glance at the map that it is but little north of Dinard, for example, which of late has become so favourite a spot for winter residence. There is more rain in the neighbourhood of Penzance than in northern France, but the atmosphere is very mild; it is absolutely sheltered from north and east winds, and Sir James Clark says that there is no place in England where you can spend longer out of doors. Living is much the same as in Newquay or Dartmouth—not so expensive as Brighton or Eastbourne. The excursions near and far are very charming, and the town is not destitute of literary attractions. Walks abound—to Newlyn and Mousehole is a very favourite one, and quite short. Most people have heard of the "Newlyn school" of artistes, who form quite a colony of themselves, and occupy something like the position of the "Barbizon school" in France. The Land's End is a longer affair, but well repays a visit. Marazion and St. Michael's Mount is easily seen, and there is a station at Marazion. At low water you cross to the mount from Marazion, but at high tide it is quite cut off, and must be reached in a boat. It strongly resembles the Michael's Mount of Normandy, but is not so impressive and much more modernised.

### Newquay has become during the last few years quite fashionable, and has lost much of its charm in consequence, but it must be admitted that the extension of the railway and the increased accommodation for visitors have greatly added to its comfort for the sojourners on its fine cliffs. It is not so warm as Penzance, being exposed to the north wind, but on that very account many would prefer it as being less enervating. There are delightful excursions to be made around, and if your pedestrian powers are but indifferent there are many short walks which show you very varied scenery. Tintage is an affair of an entire day, mostly by coach, driving straight across the moors, rather a bleak journey in winter. I made it in snow and frost the first week of January, but thoroughly enjoyed it all the same.

### Eastbourne and Brighton.

Eastbourne is by no means a warm place. It is exposed to the east wind, and is certainly cold, but bright and exhilarating. Many who are otherwise strong, but have met with a breakdown from overwork or some such cause find the air of Eastbourne most beneficial, and the numerous walks and drives in the brisk air, blowing either from the sea or the Sussex downs, acts like champagne. The same may be said of Brighton, which is less exposed to the east wind; but with many is not so well liked, being undeniably a town, and a very large one—a species of small London by the sea.

### Bournemouth will always remain a favourite place of winter residence; it has a well established reputation for suitability to those suffering from chest trouble. It still is a charming spot, but too much built over, and the owners of property, not unnaturally, have eagerly built more and more, till the lovely pine woods are fast disappearing. It is somewhat a case of killing the goose that lays the golden eggs, for it is the pines themselves that are so beneficial in pulmonary cases. Of late years immense improvements have been made in the direction of good walks for invalids. The pleasure gardens, the "invalids' walk," the arcade—a pleasant resort when the weather is threatening—to say nothing of the pier with its band, are all close at hand, and for more distant excursions there are numberless lovely spots to be seen. As for clubs their name is legion.

### Ventnor.

With regard to Ventnor and the other favoured spots in the Isle of Wight I think most people know pretty well all about them. As a place of residence the little island is expensive; it is almost always so when a place is so small. It is, indeed, so contracted that I experience a feeling of imprisonment when there, and always a perverse desire to get further afield. There seems a sense of crowding and impossibility of detaching oneself from one's fellow creatures. I suppose this feeling is somewhat general, for I heard a lady describe it as "like a tea garden—an aristocratic Rosherville, you know." Be that as it may, it has many charming spots. Ventnor seems to have established itself as *the* winter resort *par excellence*, and certainly the climate is wonderfully equable, and its close proximity to romantic Shanklin will always make it a favourite spot for residence.

### Devonshire.

I have left myself but little room to speak of beautiful Devon, and must reserve a whole article for its charms and eligible spots for winter homes, therefore I must not try to speak of them to-day.

### TRAVEL NOTES AND QUERIES.

Montreux (Alum Bay).—I think it would meet your requirements, as it is decidedly gay and lively. As to hotel and pension terms, there is nothing very low to be met with, but you can live at eight francs per day, or less if you are a party. Hotel proprietors will generally come a little way to meet you in terms.

San Remo (Publicus).—Not quite so expensive as Mentone; a trifle less sheltered, and therefore not quite so warm. From an artistic point of view there is little difference; from both places the walks and excursions are full of interest. I think I should give the preference to the excursions from San Remo, on the whole. There is one on the Italian side, and the ground is less well known. For the towns themselves, in both cases the architecture is most interesting in the old quarters, the most so in San Remo, but then that advantage is counterbalanced at Mentone by the beautiful bay and the charming effect of the varied shipping, with the old town rising behind, crowned by the church. I should go to an hotel in Mentone for two or three days, and study both places before finally deciding.

To Live in a French Family (Casino).—You ask me a difficult question. The French are very averse to taking anyone to share their family life. It is almost an impossibility to find anyone willing thus to admit an outsider. Your best chance is to apply to the various French pastors of the French Lutherans. I do not recommend this by any means, but I think it is all that can be done unless you have friends in France.

### To Nurses.

We invite contributions from any of our readers, and shall be glad to pay for "Notes on News from the Nursing World," or for articles describing nursing experiences, or dealing with any nursing question from an original point of view. The minimum payment for contributions is 5s., but we welcome interesting contributions of a column, or a page, in length. It may be added that notices of entertainments, presentations, and deaths are not paid for, but, of course, we are always glad to receive them. All rejected manuscripts are returned in due course, and all payments for manuscripts used are made as early as possible at the beginning of each quarter.

